# Recent Research and Applications of Numerical Simulation for Dynamic Response of Long-Span Bridges Subjected to Multiple Loads

**DOI:** 10.1155/2014/763810

**Published:** 2014-05-21

**Authors:** Zhiwei Chen, Bo Chen

**Affiliations:** ^1^Department of Civil Engineering, Xiamen University, Xiamen, Fujian 361005, China; ^2^Key Laboratory of Roadway Bridge and Structural Engineering, Wuhan University of Technology, Wuhan 430070, China

## Abstract

Many long-span bridges have been built throughout the world in recent years but they are often subject to multiple types of dynamic loads, especially those located in wind-prone regions and carrying both trains and road vehicles. To ensure the safety and functionality of these bridges, dynamic responses of long-span bridges are often required for bridge assessment. Given that there are several limitations for the assessment based on field measurement of dynamic responses, a promising approach is based on numerical simulation technologies. This paper provides a detailed review of key issues involved in dynamic response analysis of long-span multiload bridges based on numerical simulation technologies, including dynamic interactions between running trains and bridge, between running road vehicles and bridge, and between wind and bridge, and in the wind-vehicle-bridge coupled system. Then a comprehensive review is conducted for engineering applications of newly developed numerical simulation technologies to safety assessment of long-span bridges, such as assessment of fatigue damage and assessment under extreme events. Finally, the existing problems and promising research efforts for the numerical simulation technologies and their applications to assessment of long-span multiload bridges are explored.

## 1. Introduction

**Figure 1:**
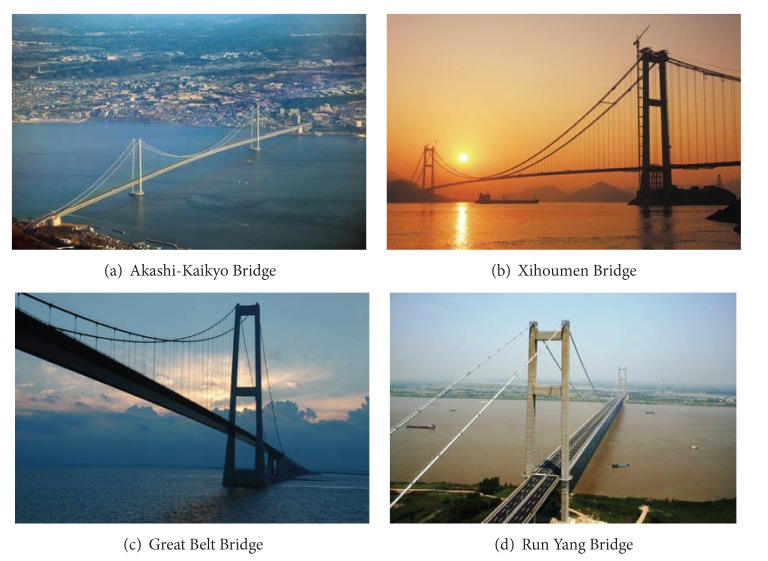
Examples of long-span bridges.

Many long-span bridges have been built throughout the world in the past few decades to meet the economic, social, and recreational needs of communities. Some of these bridges have main span lengths of more than 1000 m (see Figure 1), such as the Akashi Kaikyo Bridge (1,991 m, Japan, 1998), the Xihoumen Bridge (1,650 m, China, 2009), the Great Belt Bridge (1,624 m, Denmark, 1998), and the Run Yang Bridge (1,490 m, China, 2005). Some of them carry both road and rail traffic, such as the Tsing Ma Bridge (1,377 m, Hong Kong, 1997), the Minami Bisan-Seto Bridge (1,100 m, Japan, 1989), and the 25 de Abril Bridge (1,013 m, Japan, 1966). Most of these bridges are located in wind-prone regions, and long-span length makes them susceptible to strong crosswinds. Further, the increases in traffic volume and gross vehicle weight that accompany economic development significantly affect the local dynamic behavior of such bridges. Most of long-span bridges are multiload bridges since they are simultaneously suffering combined effects of multiple dynamic loading, such as railway, highway, and wind loading. Multiload bridges play significant roles in the entire transportation system, and thus it is critically important to protect such immense capital investments and ensure user comfort and bridge safety.

However, the strength and integrity of these bridges will decrease during the serviceability stage due to the degradation mechanisms induced by traffic, wind, temperature, corrosion, and environmental deterioration. In order to detect the abnormal changes through nondestructive testing (NDT) technology or periodical evaluation, a fundamental but critical step is to obtain dynamic responses at some critical bridge locations. The mostly concerned dynamic responses of a multiload bridge may include global response (displacement, velocity, and acceleration) and local response (acceleration and stress), which are mainly induced by traditional live load (such as highway, railway, and wind loading) or accidental live load (such as ship impact and earthquake). Structural intrinsic characteristics could be extracted from these dynamic responses (or vibration signals) to develop all sorts of vibration-based damage detection techniques. A well-known family of them is based on structural dynamic characteristics (such as frequencies, mode shapes, damping ratios, and strain mode shapes) and their derivatives [1–3]. Some damage identification approaches were proposed based on the dynamic responses of bridge structures under moving vehicle loads [4–6]. The dynamic responses of long-span bridges also could be used for structural assessment, for example, fatigue assessment at the critical locations over the service history of the bridge [7–10] and assessment of extreme events such as complex traffic congestion coupled with moderate or even strong wind [11].

Over the past decades, on-structure long-term structural health monitoring systems (SHMSs) have been implemented on long-span bridges in Europe, the United States, Canada, Japan, Korea, China, and other countries [12]. They are installed in newly constructed bridges and existing bridges for monitoring structural behavior in real time, evaluating structural performance under various loads, and identifying structural damage or deterioration [13]. To comprehensively understand the bridge performance, dynamic bridge responses are important monitoring items of structural health monitoring. Global responses (such as displacement) are measured by GPS and accelerometers [14, 15], and local responses (such as strain/stress) are normally measured in the critical bridge components and widely used for fatigue assessment [16]. Although dynamic responses have been measured for those bridges installed with SHMSs, condition evaluation based on measurement still has some limitations: (1) it is difficult to identify all of the local critical locations, and even so, it is uneconomical to monitor all critical locations in long term; (2) not every fatigue-critical location is suitable for sensor installation; (3) it is difficult to obtain measurement data in the extreme events (such as combination of traffic congestion and strong wind) which rarely happen; (4) it is hard to exactly predict the influence of possible traffic growths based on field measurement only. Integrating with numerical simulation technologies and field measurements is an alternative approach which is able to overcome the limitations of evaluation approaches only based on measurements. The information on the concerned dynamic loadings measured by the SHMS could be taken as inputs for the numerical simulation, and the computed dynamic responses could be compared with the measured ones in the validation [17].

However, numerical simulation of dynamic response of a long-span multiload bridge is not an easy job, because it requires a complex dynamic finite element model of the bridge including all important bridge components, various dynamic loading models for running trains, running road vehicles, and high winds, and interactive models between the bridge and wind, bridge and trains, and bridge and road vehicles [17]. This paper focuses on recent research and applications of numerical simulation technology for dynamic response of long-span multiload bridges. Firstly, key issues involved in dynamic response analysis of long-span multiload bridges based on numerical simulation technologies are reviewed in Section 2. The applications of newly developed numerical simulation technologies to safety assessment of long-span bridges are subsequently reviewed in Section 3. Finally, the existing problems and promising research efforts for the numerical simulation technologies and their applications to assessment of long-span multiload bridges are explored in Section 4.

## 2. Numerical Simulation: Dynamic Responses of Long-Span Multiload Bridges

For the most complex situation, a long-span multiload bridge which is located at a wind-prone region carries both railway and highway traffic, and thus the combined effect of running trains, running road vehicles, and wind is acting on the bridge. Several key issues are involved in this complicated situation, such as dynamic interaction between running trains and bridge, dynamic interaction between running road vehicles and bridge, and dynamic interaction between wind and bridge. To give a comprehensive review, the above three key issues will be individually reviewed in Sections 2.1 to 2.3 and then the dynamic interactions of wind-vehicle-bridge system as a whole are then reviewed in Section 2.4.

### 2.1. Dynamic Interaction between Trains and Bridge

#### 2.1.1. Modeling of a Cable-Supported Bridge

In early research in this area, simplified bridge models were employed to study vehicle-bridge interactions. For example, a cable-stayed bridge was simulated as a beam resting on an elastic foundation by Meisenholder and Weidlinger [18] for the dynamic analysis of cable-stayed guideways subject to track-levitated vehicles moving at high speeds. Mao [19] investigated the impact factor of a cable-stayed bridge, which was assumed to be formed of continuous elastic beams supported by intermediate elastic supports.

**Figure 2:**
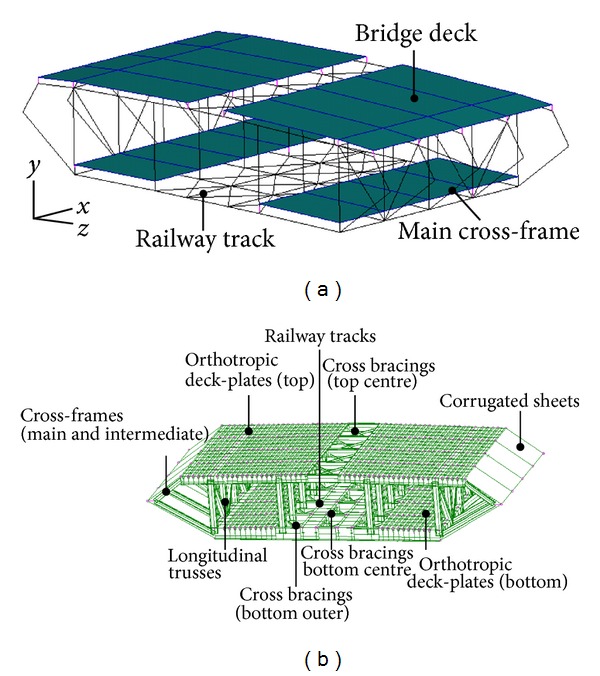
Finite element model of suspended deck module: (a) hybrid 3D bridge model [22]; (b) full 3D model [25].

More recently, with the development of finite element (FE) technology, it has become common practice to use a computer software package to establish a finite element model (FEM) of a cable-supported bridge. This technology establishes an accurate bridge model that takes into account the geometric nonlinear behavior of a cable-supported bridge. To make the bridge model close to the realistic bridge in terms of its dynamic properties, the modal frequencies and shapes determined by dynamic tests are used for further model validation or updating. Many FEMs of cable-supported bridges have been established for the analysis of train-bridge interactions. The Tsing Ma Suspension Bridge in Hong Kong can be used as an example to illustrate the various bridge models that have been established for analysis. The first generation of Tsing Ma Bridge model was a spinal beam model [20] in which the hybrid steel deck was represented by a single beam with equivalent cross-sectional properties, two bridge towers made of reinforced concrete that were modeled by three-dimensional Timoshenko beam elements, and cables and suspenders that were modeled by cable elements to account for geometric nonlinearity due to cable tension. The model was validated by comparing it with measurements of the first 18 modal frequencies and shapes of the actual bridge. Using this model, Guo et al. [21] predicted the dynamic displacement and acceleration responses of coupled train and bridge systems in crosswinds. However, they modeled the bridge deck as a simplified spine beam of equivalent sectional properties and were thus unable to capture the local stress and strain behavior of the bridge. A second-generation bridge model (hybrid 3-dimensional model) was established to overcome this weakness [22]. The modeling work is based on the previous model developed by Wong [23]. In this model, 15,904 beam elements were used to model the bridge deck to closely replicate the geometric details of the complicated deck in reality (see Figure 2(a)). The railway beams and rails were modeled by beam elements to allow the accurate computation of the contact forces between the bridge and railway vehicle. The deck-plates carrying the road vehicles were modeled by plate elements to allow the accurate computation of the contact forces at the contact points between the road surface and the vehicle tires. The bridge deck was modeled to closely replicate the geometric details of the complicated deck which is required for calculation of the action of the wind forces. The bridge model was also updated using the first 18 measured natural frequencies and mode shapes. Based on this model, Xu et al. [24] computed the stress and acceleration responses of local critical components under running trains, and Chen et al. [17] computed dynamic stress response induced by railway, highway, and wind loading.

However, the hybrid 3D model is still not fine enough for criticality analysis of bridge structures which requires results at strain/stress level, especially for some bridge details. For example, the orthotropic decks (steel deck-plates supported by U-shape troughs) were modeled by plate elements with equivalent depths so that the measured results from strain gauges at the surfaces of deck-plates or U-shape troughs had no counterparts in computation results. Therefore, Duan et al. [25] established the third-generation bridge model (full 3D model) for performance evaluation at stress/strain level (see Figure 2(b)). In this model, the major structural components were modeled in detail and the connections and boundary conditions are modeled properly, which results in about half million elements for the complete bridge model. The strain/stress responses induced by a train passing through the bridge were calculated by static influence line method and compared with measured results in the calibration.

Although full 3D bridge model provides the possibility for exact stress analysis at the local components, large computational efforts are needed for the refined section model with complicated structural details. Li et al. [26] proposed a multiscale FE modeling strategy for long-span bridges. The global structural analysis was carried out using the beam element modeling method at the level of a meter. The local detailed hot-spot stress analysis was carried out using shell or solid elements at the level of a millimeter. Based on this model, the global dynamic response of the bridge and local damage accumulation of two typical weld details of the bridge under traffic loading were numerically analyzed. Multiscale FE modeling scheme was also proposed by Zhang et al. [27] based on the equivalent orthotropic modeling method (EOMM). Bridge details with multiple stiffeners were modeled with shell elements using equivalent orthotropic materials. Based on this model, Zhang et al. [10] computed the dynamic stress responses of long-span bridges under combined dynamic loads from winds and road vehicles.

#### 2.1.2. Modeling of Trains

Previously, running vehicles were commonly modeled as a series of moving forces, either due to limits on computational capacity or because it is easier to find the analytical solutions in many cases [28–37]. This treatment neglects the effect of interactions between the bridge and running vehicles. For this reason, the moving load model is suitable only for the case in which the mass of the vehicle is small relative to that of the bridge or when the vehicle response is not of interest [38]. For cases in which the inertia of the vehicle cannot be regarded as small, a moving mass model should be adopted instead [39–42]. More recently, the emergence of high-performance computers and advances in computer technology has made it feasible to more realistically model the dynamic properties of the various components of moving vehicles [43–48].

**Figure 3:**
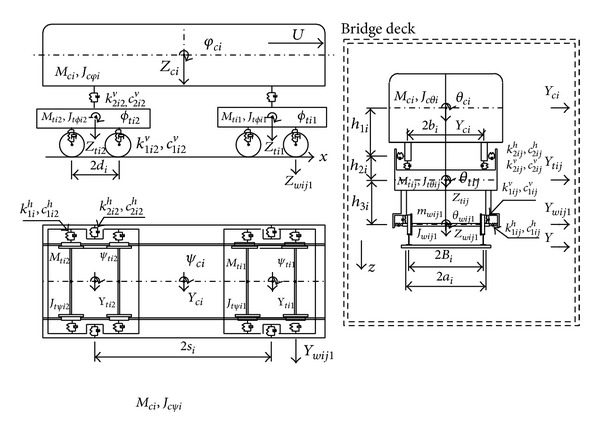
Dynamic model of a railway vehicle [51].

In a more sophisticated railway vehicle model, the suspension mechanisms are modeled by springs, the damping effect of the suspension systems and air-cushion by dashpots, and the energy dissipating effect of the interleaf mechanism by frictional devices. Using this technique, a tractor-trailer is represented as two discrete masses, each of which is supported by two sets of springs and dashpots or frictional devices [38]. To represent the various dynamic properties of railway vehicles, vehicle models that contain dozens of degrees of freedom (DOFs) have been devised and used by [49–52]. To investigate the dynamic interaction between a long suspension bridge and running trains, Xia et al. [51] considered a train composed of a sequence of identical railway vehicles. Each railway vehicle was assumed to consist of a rigid car body resting on front and rear bogies, with each bogie supported by two wheelsets (see Figure 3). Five DOFs were assigned to the car body and to each bogie to account for vertical, lateral, rolling, yawing, and pitching motions. In contrast, only three DOFs were assigned to each wheelset to account for vertical, lateral, and rolling motions.

**Figure 4:**
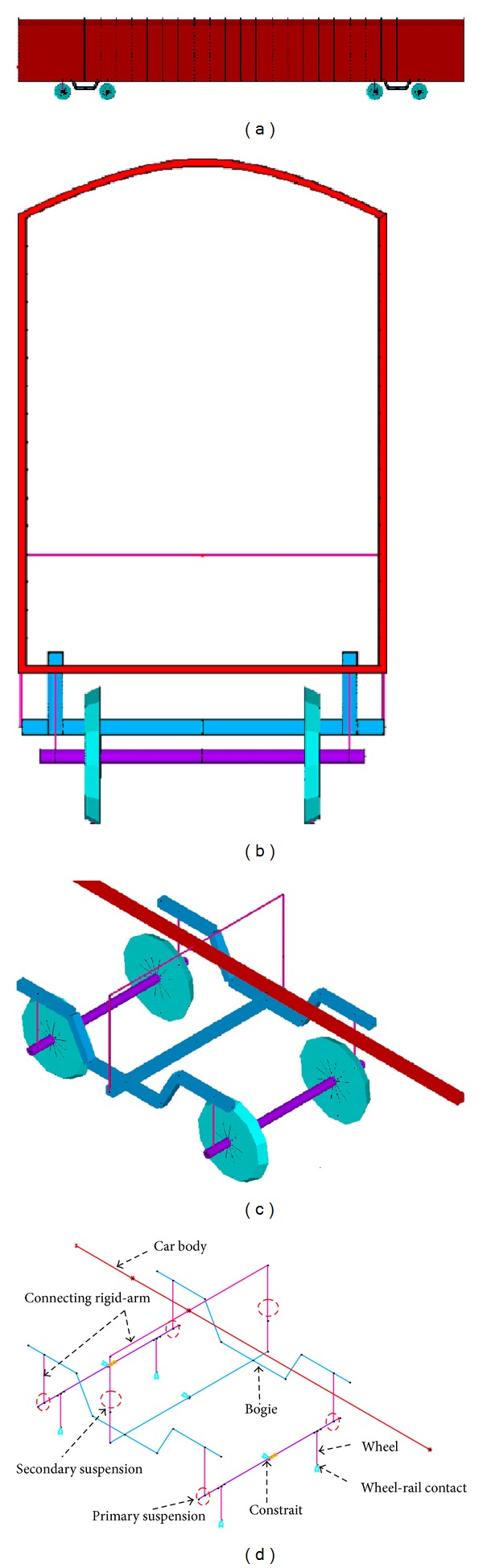
Finite element model of a railway vehicle: (a) elevation view; (b) side view; (c) isometric view; (d) model details [52].

Many vehicle models have been established for vehicle-bridge interaction analysis. In most of these studies, the equations of motion of the vehicles were derived analytically. However, a great inconvenience of this method is that the equations of motion of the whole vehicle-bridge system must be rederived if the vehicle type is changed. Furthermore, it is very difficult to derive the equation of motion for a complex vehicle model containing a large number of DOFs, such as the articulated components of a TGV train with an 85-DOF dynamic system [53]. General commercial FE software has recently been adopted to make vehicle modeling more easily applicable for different vehicle types [54]. Li et al. [55] described a four-step procedure for modeling a four-axle railway vehicle by beam elements: (1) the nodes and elements for the car body, bogies, and wheelsets, respectively, are defined by using beam elements so that the spatial geometric configuration of each component can be built (see Figure 4); (2) sectional properties and material properties are assigned to each beam element; (3) rigid-arms and suspension units (systems) are used to connect the three components; (4) constraints are assigned to form a complete finite element model of the vehicle.

#### 2.1.3. Modeling of Rail Irregularities

Track irregularities represent an important source of excitation for bridges during the passage of railway vehicles. Track irregularities may occur as a result of initial installation errors, the degradation of support materials, or the dislocation of track joints. Four geometric parameters can be used to quantitatively describe rail irregularities: the vertical profile, cross level, alignment, and gauge [49, 50, 56]. Vertical profile and cross level irregularities chiefly influence the vertical vibrations of vehicles and of the bridge, whereas alignment, gauge, and cross level irregularities initiate horizontal transverse vibrations of vehicles and the bridge and also the torsional movement of the bridge [57]. Track irregularities may be periodic or random. Random irregularities are due to wear, clearance, subsidence, and insufficient maintenance. For engineering applications, random irregularities can be approximately regarded as stationary and ergodic processes that can be generated from measured results or simulated by numerical methods. Several numerical methods have been proposed for the simulation of random rail irregularities, such as the trigonometry series, white noise filtration, autoregressive (AR), and power spectral density (PSD) sampling methods. Among these methods, the PSD sampling method has been widely adopted due to its high computational accuracy. The lateral and vertical irregularities could be all assumed to be zero-mean stationary Gaussian random processes and expressed through the inverse Fourier transformation of a PSD function [58]:

(1) ys(x)=∑k=1N2S(fk)Δfcos⁡(2πfkx+θk),

where *S*(*f*) is the PSD function; *f*_*k*_ = *f*_l_ + (*k* − 1/2)Δ*f*; Δ*f* = (*f*_*u*_ − *f*_l_)/*N*; *f*_*u*_ and *f*_*l*_ are the upper and lower cutoff frequencies, respectively; and *θ*_*k*_ is the random phase angle uniformly distributed between 0 and 2*π*. Rail irregularity in railway engineering is commonly represented by a one-sided PSD function.

The PSD functions of rail irregularities have been developed by different countries. Based on the PSD functions of rail irregularities developed by the Research Institute of the China Railway Administration, Zhai [59] expressed all rail irregularities using the unified rational formula as follows:

(2) S(f)=A(f2+Bf+C)f4+Df3+Ef2+Ff+G,

where *f* = 1/*η* (m^−1^) is the spatial frequency in cycle/m (*η* is the wavelength) and *A* to *G* are the parameters recommended by Zhai [59] specifically for vertical and lateral rail irregularities.

#### 2.1.4. Solution Methods

The dynamic analysis of vehicle-bridge coupled system requires two sets of equations of motion for the bridge and vehicles, respectively. These describe the interaction or contact forces at the contact points of the two subsystems. Because the contact points move from time to time, the system matrices are generally time dependent and must be updated and factorized at each time step. The various solution methods can be generalized into two groups according to whether or not an iterative procedure is needed at each time step.

The first group of methods solves the equations of motion of a coupled vehicle-bridge system at each time step without iteration. This approach has been widely used in coupled vehicle-bridge analysis [51, 53, 60–69]. These methods have good computational stability and are convenient for dealing with vehicle-bridge interaction problems when the vehicle model is relatively simple. The main disadvantage is that the equations of motion of the coupled system are time dependent, and thus the characteristic matrices must be modified at each time step. In addition, the equations of motion of the coupled vehicle-bridge system become very difficult to determine if nonlinear wheel-rail contacts and nonlinear vehicle models are considered.

The second group of methods solves the equations for the vehicles and bridge separately and requires an iterative process to obtain convergence for the displacements of the vehicles and bridge at all contact points. Given that the conditions of wheel-rail contact geometry and contact forces are rather complex, a stable integration method adopting a small time interval is needed for obtaining the convergence of vehicle and bridge subsystems at the contact points in each time step. Many studies have applied this type of method to investigate vehicle-bridge interactions [70–76]. The advantage of these methods is that the dynamic property matrices in the two sets of equations of motion remain constant, which is convenient for the consideration of nonlinear vehicle-bridge interactions and nonlinear vehicle models [55]. However, in engineering applications, the iterative convergence is a critical problem with this type of method. The low convergence rate and occasional divergence of the solution have also been noted [77]. Li et al. [55] investigated the performance of these iterative schemes using the Wilson-**θ** method, Newmark-**β**method, and an explicit integration method proposed by Zhai [59] and found that the latter gave a much higher convergence rate than the former two methods.

Most of the above methods solved the equations of motion of a coupled vehicle-bridge system using the nonjump model, which assumes that the moving vehicle traveling along the bridge is always in contact with the rails, no matter what the sign is of the contact forces. This is not always true in view of the physics of the moving vehicle which simply sits on the upper surfaces of the rails. The interaction forces between the moving vehicle and the bridge depend on the motions of the vehicle, the flexibility of the bridge, and the track irregularities. Li et al. [55] utilized a jump model to solve vehicle-bridge interaction problem using a noniterative Runge-Kutta method and found that the acceleration responses of the car body using the wheel-jump model are smaller than those using the wheel nonjump model when the vehicle speed exceeds 300 km/hr. Antolin et al. [78] proposed a nonlinear wheel-rail interaction model which considers nonlinear wheel-rail contact forces in the interaction as well as realistic wheel and rail profiles and applied it for analysis of dynamic interaction between high speed trains and bridges.

### 2.2. Dynamic Interaction between Road Vehicles and Bridge

Section 2.1 gave a detailed literature review of the dynamic interactions between trains and bridges. As there are some fundamental differences between trains and road vehicles, this section reviews the modeling of road vehicles, the simulation of road vehicle flow, and the modeling of road surface roughness.

#### 2.2.1. Modeling of Road Vehicles

**Figure 5:**
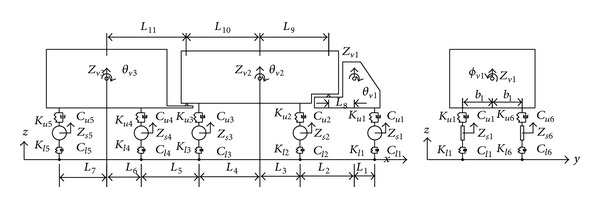
Dynamic model of a tractor-trailer [79].

**Figure 6:**
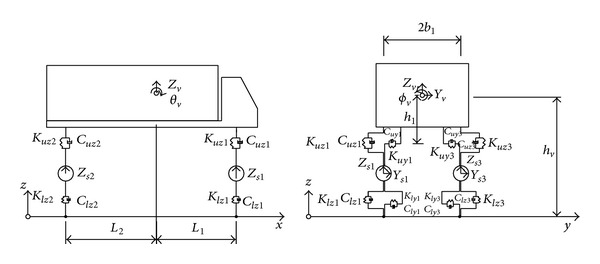
Dynamic model of a high-sided road vehicle [80].

To analyze the dynamic interaction between a bridge and running road vehicles, a model of road vehicles must be established. A sophisticated road vehicle model is required to make the simulation as realistic as possible. A road vehicle is modeled as a combination of several rigid bodies, each of which is connected by a set of springs and dashpots which model the elastic and damping effects of the tires and suspension systems, respectively. There are various configurations of road vehicles, such as a tractor and trailer with different axle spacing. Road vehicle models that contain several DOFs have been devised for vehicle-bridge interaction analysis. For example, Guo and Xu [79] modeled a 17-DOF four-axle heavy tractor-trailer vehicle (see Figure 5) to investigate the interaction between vehicles and a cable-stayed bridge. A total of three DOFs were assigned to rigid bodies representing either the tractor or the trailer to account for vertical, rolling, and pitching motions. Only one DOF was assigned to the rigid body representing the axle set moving in the vertical direction. Different vehicle models are adopted in wind-vehicle-bridge interaction analyses. Xu and Guo [80] modeled a 13-DOF two-axle road vehicle (see Figure 6) for the dynamic analysis of a coupled road vehicle and bridge system under turbulent wind. Five DOFs were assigned to the vehicle body with respect to its center of gravity to account for vertical, lateral, rolling, yawing, and pitching motions, and two DOFs were assigned to the front and rear axle sets to account for motions in the vertical and lateral direction. More DOFs are needed to account for lateral crosswinds.

#### 2.2.2. Simulation of Road Vehicle Flow

On long-span bridges there is a high probability of the simultaneous presence of multiple road vehicles, including heavy trucks. This may lead to larger amplitude stress responses and greater fatigue damage of the local bridge components than would be the case with only one road vehicle. The simulation of road vehicle flow is thus important in the analysis of the dynamic interaction between road vehicles and bridges. Rather simple patterns of road vehicle flow have been assumed in most vehicle-bridge coupled dynamic analyses [79, 81, 82] in which either one or several vehicles are distributed on the bridge in an assumed (usually uniform) pattern. Obviously, such assumptions do not represent actual road traffic conditions. Recently, Chen and Wu [83] modeled the stochastic traffic load for a long-span bridge based on the cellular automaton (CA) traffic flow simulation technique. In this study, they simulated a complicated road vehicle flow on long-span bridges in terms of vehicle number, vehicle type combination, and driver operation characteristics, such as lane changing, acceleration, or deceleration.

#### 2.2.3. Modeling of Road Surface Roughness

Road surface roughness is an important factor that greatly affects vehicle-bridge interactions. Paultre et al. [84] pointed out that road surface or pavement roughness can significantly affect the impact response of a bridge. The roughness or surface profile depends primarily on the workmanship involved in the construction of the pavement or roadway and how it is maintained, which, although random in nature, may contain some inherent frequencies [38]. In most cases, surface roughness, which is three-dimensional in reality, is often approximated by a two-dimensional profile. To account for its random nature, the road profile can be modeled as a stationary Gaussian random process and derived using a certain power spectral density function. Other methods similar to this have been widely adopted by researchers studying vehicle-induced bridge vibration [65, 70, 71, 85–90]. Dodds and Robson [91] developed power spectral density functions that were later modified and used by Wang and Huang [87] and Huang et al. [92]. This approach was also adopted by literatures [79, 81] in their dynamic analyses of coupled vehicle-bridge and wind-vehicle-bridge systems.

### 2.3. Dynamic Interaction between Wind and Bridge

When a long-span cable-supported bridge is immersed in a given flow field, the bridge will be subject to mean and fluctuating wind forces. To simulate these forces, a linear approximation of the time-averaged static and time-varying buffeting and self-excited force components must be formulated [93, 94]. As dynamic bridge responses are of concern in this study, only buffeting and self-excited forces are considered and reviewed in this section.

#### 2.3.1. Buffeting Forces

Buffeting action is a random vibration caused by turbulent wind that excites certain modes of vibration across a bridge depending on the spectral distribution of the pressure vectors [95]. Although the buffeting response may not lead to catastrophic failure, it can lead to structural fatigue and affect the safety of passing vehicles [96]. Hence, buffeting analysis has received much attention in recent years in research into the structural safety of bridges under turbulent wind action [81, 95, 97–102].

By assuming no interaction between buffeting forces and self-excited forces and using quasi-steady aerodynamic force coefficients, the buffeting forces per unit span **F**_bf_^ei^ on the *i*th section of a bridge deck can be expressed as [103]

(3) Fbfei=[0LbfeiDbfeiMbfei00]=12ρUi2BiLi[00χLbu(2CLiUi)χLbw(CLi′+CDiUi)χDbu(2CDiUi)χDbw(CDi′Ui)χMbu(2CMiUi)BiχMbw(CMi′Ui)Bi0000] ×{uiwi},

where *D*_bf_^ei^, *L*_bf_^ei^, and *M*_bf_^ei^ are the buffeting drag, lift, and moment, respectively; *u*_*i*_ and *w*_*i*_ are the horizontal and vertical components, respectively, of fluctuating wind at the *i*th section; *ρ* is the air density; *U*_*i*_ is the mean wind speed at the *i*th section of the bridge deck; *B*_*i*_ and *L*_*i*_ are the width and length of the *i*th bridge section; *C*_*Di*_, *C*_*Li*_, and *C*_*Mi*_ are the drag, lift, and moment coefficients, respectively, of the *i*th bridge segment; *C*_*Di*_′ = *dC*_*Di*_/*dα*′, *C*_*Li*_′ = *dC*_*Li*_/*dα*′, and *C*_*Mi*_′ = *dC*_*Mi*_/*dα*′; *α*′ is the angle of attack of a normal wind incident on the horizontal plane of the deck; and *χ*_*D*_bu__, *χ*_*D*_bw__, *χ*_*L*_bu__, *χ*_*L*_bw__, *χ*_*M*_bu__, and *χ*_*M*_bw__ are the aerodynamic transfer functions between the fluctuating wind velocities and the buffeting forces.

It can be found from this equation that a series of time histories of fluctuating wind velocity {*u*_*i*_,*w*_*i*_}^*T*^ in the longitudinal and vertical directions at various points along the bridge deck is needed to carry out a detailed buffeting analysis. To simulate the stochastic wind velocity field, the fast spectral representation method proposed by Cao et al. [104] that is based on the spectral representation method developed by Shinozuka and Jan [105] is often adopted. This method rests on the assumptions that (1) the bridge deck is horizontal at the same elevation, (2) the mean wind speed and wind spectra do not vary along the bridge deck, and (3) the distance between any two successive points where wind speeds are simulated is the same. The time histories of the along-wind component *u*(*t*) and the upward wind component *w*(*t*) at the* j*th point can be generated using the following equations [104]:

(4a) uj(t)=2(Δω)∑m=1j∑k=1NfSuu(ωmk)×Gjm(ωmk)cos⁡(ωmkt+φmk),

(4b) wj(t)=2(Δω)∑m=1j∑k=1NfSww(ωmk)×Gjm(ωmk)cos⁡(ωmkt+φmk),

(4c)  Gjm(ω)={0,when  1≤j<m≤nC|j−m|,when  l=1,  m≤j≤nC|j−m|(1−C2),when  2≤m≤j≤n,

(4d)  C=exp⁡(−λωmkΔ2πU); Δ=Lnp−1,

(4e)  ωmk=(k−1)Δω+mnΔω, (k=1,2,…,Nf),

where Δ*ω* = *ω*_up_/*N*_*f*_ is the frequency interval between the spectral lines; *N*_*f*_ is the total number of frequency intervals; *ω*_up_ is the upper cutoff frequency; *n* is the total number of points at which wind speeds are simulated; *S*_uu_ and *S*_ww_ are the along-wind and vertical wind spectrum, respectively; *φ*_lm_ is a random variable that is uniformly distributed between 0 and 2*π*; *L* is the span length; and *λ* is a parameter that usually falls between 7 and 10.

**Figure 7:**
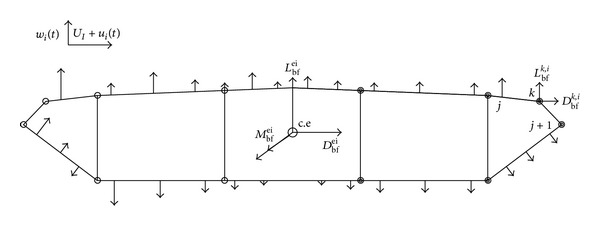
Buffeting wind pressures and buffeting forces at nodes [22].

In reality, the equivalent buffeting forces in (3) are actually associated with the spatial distribution of the wind pressures on the surface of the bridge deck. Ignoring the spatial distribution or aerodynamic transfer function of the buffeting forces across the cross-section of the bridge deck may have a considerable impact on the accuracy of buffeting response predictions. Furthermore, the local structural behavior of the bridge deck associated with local stresses and strains, which are prone to causing local damage, cannot be predicted directly by the current approaches based on equivalent buffeting forces. In this regard, Liu et al. [22] proposed an approach to consider the spatial distribution of buffeting forces on a bridge deck structure based on wind pressure distributions from wind tunnel tests (see Figure 7).

#### 2.3.2. Self-Excited Forces

In addition to buffeting action, flutter instability caused by self-excited forces induced by wind-structure interactions is an important consideration in the design and construction of long-span suspension bridges [96], because the additional energy injected into the oscillating structure by the aerodynamic forces increases the magnitude of vibration, sometimes to catastrophic levels [95]. The self-excited forces on a bridge deck are attributable to the interactions between wind and the motion of the bridge. When the energy of motion extracted from the flow exceeds the energy dissipated by the system through mechanical damping, the magnitude of vibration can reach catastrophic levels [106]. Expressing self-excited forces in the form of indicial functions was first suggested by Scanlan [94]. Based on the assumption that self-excited forces are generated in a linear fashion, Lin and Yang [107] simplified the self-excited forces acting on a bridge deck and expressed them in terms of convolution integrals between the bridge deck motion and the impulse response functions:

(5a) feDse(t) =12ρU2∫−∞t[IDh(t−τ)he(τ)+IDq(t−τ)qe(τ)+BIDθ(t−τ)θe(τ)]dτ,

(5b) feLse(t) =12ρU2∫−∞t[ILh(t−τ)he(τ)+ILq(t−τ)qe(τ)+BILθ(t−τ)θe(τ)]dτ,

(5c) feMse(t) =12ρU2∫−∞t[BIMh(t−τ)he(τ)+BIMq(t−τ)qe(τ)+B2IMθ(t−τ)θe(τ)]dτ,

where *I*_*ψ*_  (*ψ* = *Dh*, *Dq*, *Dθ*, *Lh*, *Lq*, *Lθ*, *Mh*, *Mq*, *Mθ*) is the impulse function of the self-excited forces, in which *ψ* represents the corresponding force components and *h*_*e*_, *q*_*e*_, and *θ*_*e*_ are the equivalent vertical, lateral, and torsional displacements, respectively, at the center of elasticity of the bridge deck section. The relationship between the aerodynamic impulse functions and flutter derivatives can be obtained by taking the Fourier transform of (7) [98]:

(6) I−Dh(ω)=K2(P6∗+iP5∗),  I−Dq(ω)=K2(P4∗+iP1∗),I−Dθ(ω)=K2(P3∗+iP2∗),I−Lh(ω)=K2(H4∗+iH1∗),  I−Lq(ω)=K2(H6∗+iH5∗),I−Lθ(ω)=K2(H3∗+iH2∗)I−Mh(ω)=K2(A4∗+iA1∗),  I−Mq(ω)=K2(A6∗+iA5∗),I−Mθ(ω)=K2(A3∗+iA2∗),

where the overbars denote the Fourier transform operation; the terms containing *i* represent imaginary parts; *P*_ψ_*, *H*_ψ_*, and *A*_ψ_*  (*ψ* = 1,2,…, 6) are dimensionless flutter derivatives obtained from wind tunnel tests; *K* = *ωB*/*U* is the reduced frequency; and *ω* is the circular frequency of vibration.

According to classical airfoil theory, the impulse functions can reasonably be approximated by a rational function [108]:

(7) $$\begin{array}{c} \overset{-}{I}\left(\omega \right)=\left[C_{1}+iC_{2}\frac{2\pi }{\nu }+\overset{m}{\underset{l=1}{\sum }}C_{l+2}\frac{4\pi^{2}+i2\pi d_{l+2}\nu }{d_{l+2}^{2}v^{2}+4\pi^{2}}\right], \end{array}$$

where the value of *m* determines the level of accuracy of the approximation; *C*_1_, *C*_2_, *C*_*l*+2_, and *d*_*l*+2_(*l* = 1, 2, …, *m*) are the frequency independent coefficients; and *ν* = 2*π*/*K* is the reduced mean wind velocity. By equating the real and imaginary parts in the comparison of (8) and (9), the relationship between the dimensionless flutter derivatives and the coefficients *C*_1_^*ψ*^, *C*_2_^*ψ*^, *C*_*l*+2_^*ψ*^, and *d*_*l*+2_^*ψ*^  (where *ψ* = * Dh*,* Dq*, *Dθ*,* Lh*,* Lq*, *Lθ*,* Mh*,* Mq*, *Mθ* and *l* = 1, 2, …, *m*) can be established. These coefficients are determined by using the nonlinear least-squares method to fit the measured flutter derivatives at different reduced frequencies. The expression of the aerodynamic impulse functions in the time domain can be obtained by taking the inverse Fourier transform of the impulse functions. By substituting the related impulse response functions into (5b), the self-excited lift force at the *i*th section of bridge deck can then be derived as

(8) feL,ise =12ρaUi2Bi{C1,iLθθi(t)+C2,iLθ(BiUi)θ˙i(t)+C3,iLθ(BiUi)θ¨i(t)+∑l=1mCl+3,iLθ×∫−∞tθ˙i(t)exp⁡[−dl+3,iLθUiBi(t−τ)]dτ}  +12ρaUi2{C1,iLhhi(t)+C2,iLh(BiUi)h˙i(t) +C3,iLh(BiUi)h¨i(t)+∑l=1mCl+3,iLh ×∫−∞th˙i(t)exp⁡[−dl+3,iLhUiBi(t−τ)]dτ}  +12ρaUi2{C1,iLqqi(t)+C2,iLq(BiUi)q˙i(t) +C3,iLq(BiUi)q¨i(t)+∑l=1mCl+3,iLq×∫−∞tq˙ψ(t)exp⁡[−dl+3,iLpUiBi(t−τ)]dτ}.

In practice, the terms *C*_3,*i*_^*Lθ*^, *C*_3,*i*_^*Lh*^, and *C*_3,*i*_^*Lq*^, which are related to additional aerodynamic masses, are normally neglected, and the value of *m* is often taken as 2 [101]. Similar formulations for self-excited drag and moment can be derived with analogous definitions. The self-excited forces at the *i*th node of the bridge deck can thus be expressed as

(9) $$\begin{array}{c} F_{\text{se}}^{\text{ei}}=E_{\text{ei}}X_{\text{ei}}+G_{\text{ei}}{\dot{X}}_{\text{ei}}+{\hat{F}}_{\text{se}}^{\text{ei}}, \end{array}$$

where

(10) Xei={0heiqeiθei00},Eei=12ρU−i2[0000000C1,iLhC1,iLqBiC1,iLθ000C1,iDhC1,iDqBiC1,iDθ000BiC1,iMhBiC1,iMqBi2C1,iMθ00000000000000],Gei=12ρU−i2Bi[0000000C2,iLhC2,iLqBiC2,iLθ000C2,iDhC2,iDqBiC2,iDθ000BiC2,iMhBiC2,iMqBi2C2,iMθ00000000000000]F^seei=[0L^seeiD^seeiM^seei00]=[0∑l=12Cl+3,iLqVl+3,iLq+∑l=12Cl+3,iLhVl+3,iLh+∑l=12Cl+3,iLθVl+3,iLθ∑l=12Cl+3,iDqVl+3,iDq+∑l=12Cl+3,iDhVl+3,iDh+∑l=12Cl+3,iDθVl+3,iDθ∑l=12Cl+3,iMqVl+3,iMq+∑l=12Cl+3,iMhVl+3,iMh+∑l=12Cl+3,iMθVl+3,iMθ00],

where *V*_l+3,*i*_^*ψ*^  (*ψ* = * Dh*,* Dq*, *Dθ*,* Lh*,* Lq*, *Lθ*,* Mh*,* Mq*) are the convolution integrations of the *i*th node and can be calculated using a recursive algorithm. For example,

(11) V4,iLθ(t)=∫−∞tθ˙i(t)exp⁡[−d4,iLθUiBi(t−τ)]dτ≈exp⁡[−d4,iLθUiBiΔt][V4,iMθ(t−Δt)+Δtθ˙i(t−Δt)].

**Figure 8:**
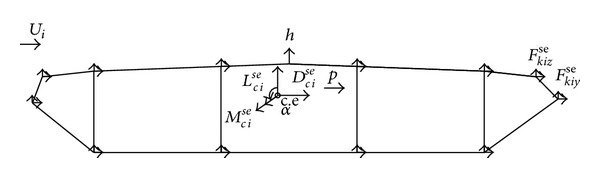
Self-excited forces at the centre of elasticity and at the nodes in the *i*th deck section [22].

The self-excited forces expressed by (9) relate to the center of elasticity of the *i*th deck section. Hence, the force model must be distributed to the nodal points of the section. A distribution based on the rigid body motion relationships between the motions at the nodal point and those at the center of elasticity of the deck section [109] was applied by Liu et al. [22]. In this study, by applying the virtual work principle, the self-excited forces at the center of elasticity of the given section were distributed to all nodes (see Figure 8).

### 2.4. Dynamic Interactions in a Wind-Vehicle-Bridge System

When trains and road vehicles are running on long-span bridges under crosswinds, complicated dynamic interactions occur among the trains, road vehicles, cable-supported bridge, and wind. The buffeting response of the bridge due to crosswind is superimposed on the dynamic response of the bridge due to railway and road vehicles. The large vibration of the bridge will in turn considerably affect the safety and ride comfort of the drivers of the road vehicles. Thus, the dynamic responses of a coupled vehicle-bridge system under crosswinds are of great concern to both engineers and researchers.

Detailed reviews of the dynamic interactions between trains and bridges, between road vehicles and bridges, and between wind and bridges have been given in the previous sections. However, the interaction between wind and vehicles must also be taken into account in a coupled wind-vehicle-bridge analysis. Many studies have investigated wind-vehicle interactions in the past few decades. Balzer [110] developed a theory to estimate the aerodynamic forces on a moving vehicle using Taylor's hypothesis of “frozen turbulence.” For engineering applications, Cooper [111] proposed the power spectral density (PSD), square-root coherence function, phase-lag function, and aerodynamic admittance function to model the unsteady side forces on a moving vehicle and laid down the foundations for investigating the effects of wind on a moving vehicle in the frequency domain. Baker developed a theoretical model that describes the dynamics of vehicles in crosswinds in the time domain [112, 113], which was later extended to include driver behavior [114]. Baker [115, 116] further investigated both the steady and unsteady aerodynamic forces acting on a variety of vehicles and carried out extensive studies of the interaction between aerodynamic forces and moving vehicles. These approaches have all been applied in coupled vehicle-bridge analysis. For example, Xu et al. [101] simulated the aerodynamic wind forces acting on running road vehicles using the quasi-steady approach, and Xu and Ding [117] derived and simulated the steady and unsteady aerodynamic forces acting on a moving railway vehicle in crosswinds in the time domain.

Based on these separate studies on the various types of dynamic interactions among wind, vehicles (trains or road vehicles), and long-span bridges, several researchers in the last decade have examined the wind-vehicle-bridge coupled system as a whole. For instance, studies have been carried out on coupled road vehicle and cable-stayed bridge systems [81, 82, 118] and on coupled train and cable-supported bridge systems in crosswinds [101, 117, 119–121]. In the recent years, several new advances have been made both in numerical simulation technologies and in wind tunnel measurements. Chen et al. [17] proposed a wind-vehicle-bridge framework which enables considering the dynamic effects induced by simultaneous actions of railway, highway, and wind loading, and it was applied to analyze dynamic stress of long suspension bridges. Li et al. [122] extended the wind-vehicle-bridge couple analysis to the case of two trains meeting on a long-span suspension bridge. Chen and Wu [118] proposed a semideterministic analytical model which is able to consider dynamic interactions between the bridge, wind, and stochastic “real” traffic. Based on the wind tunnel tests, Dorigatti et al. [123] measured crosswind loads on high-sided vehicles over long-span bridges, taking three different vehicles (van, double deck bus, and lorry) and two different bridge deck configurations into consideration. Zhu et al. [124] investigated aerodynamic coefficients of road vehicles by adopting different road vehicles types, wind directions, and vehicle positions. Li et al. [122] studied the effects of sudden changes of wind loads as the train passing through a bridge tower or two trains passing each other by using the wind tunnel test rig with moving train models. Han et al. [125] developed an experimental setup for measuring the aerodynamic characteristics of vehicles and the bridge in wind tunnel and then investigated the influences of parameters adopted in the tests.

## 3. Applications of Simulation Technology to Bridge Assessment 

After reviewing the key issues of numerical simulations for dynamic response of long-span multiload bridges, this section will review the engineering applications of the newly developed technologies to safety assessment of long-span bridges, such as assessment of fatigue and assessment under extreme events.

### 3.1. Assessment of Fatigue Damage

Steel structures are widely used in long-span bridges. Research by the ASCE [126] indicates that 80–90% of failures in steel structures are related to fatigue and fracture. Several disasters resulting from fatigue-induced bridge failure have occurred in history. For instance, 46 people died in the collapse of the Silver Bridge (USA, 1967), and 32 people lost their lives in the collapse of the Sungsoo Grand Bridge (South Korea, 1994). These disasters teach us that fatigue is an important aspect of the safety of steel bridges, and action should be taken to prevent similar fatigue-induced bridge failures. In the past few decades, fatigue assessment of steel bridges has attracted increasing attention from both governments and bridge engineers, and relevant provisions have been stipulated in several codes and standards [127–130].

It has great advantages to evaluate fatigue damage of long-span bridges based on numerical simulation, especially for a multiload bridge which suffers multiple types of dynamic loading, such as railway, highway, and wind loading. Different from sudden structural damage, fatigue damage accumulates with load-induced dynamic stress (or stress fluctuation) over the service life of a bridge lasting for more than 100 years. The increase in traffic volume and gross vehicle weight that accompany economic development is very likely to happen in the long period. Numerical simulation technology can be an ideal tool to study influences of traffic growth patterns to fatigue damage of bridge. In addition, slender long-span bridges built in wind-prone regions also suffer from considerable wind induced vibration, which appears within a wide range of wind speeds and lasts for almost the whole design life of the bridge. Given the simultaneous presence of multiple vehicles and wind, it is necessary to consider combined effects of traffic loading (railway and/or highway loading) and wind loading in the fatigue assessment. Since multiple loading is concerned in a long time period, there are a large number of loading combinations for multiple loading in different intensities. It is almost unavailable for field measurement to obtain such complete information, but numerical simulation could be a good choice to determine dynamic responses of a long-span bridge under multiple loading.

A number of structural health monitoring systems (SHMSs) have been installed on numerous recently built long-span bridges, and a variety of sensors are used for monitoring bridge loadings (e.g., traffic, wind, and earthquakes) and conditions (including global and local responses) to ensure bridge safety and user comfort under in-service conditions. Well-known examples include Tsing-Ma Bridge in Hong Kong, Akashi Kaikyo Bridge in Japan, Binzhou Yellow River Bridge in China, and Jindo Bridge in Korea. Integration of numerical simulation technologies and measurement data of structural health monitoring systems (SHMSs) installed on long-span bridges will make the fatigue assessment more reliable for several reasons: (1) it is a perfect validation by using field measurement data of the different types of loading as input of numerical simulation and the measured dynamic responses for comparison with the computed ones; (2) a large number of measured loading data could be used to establish loading databases or probabilistic models of different loads.

In the recent years, several researchers [7–10] applied the newly developed numerical simulation technologies to fatigue assessment of long-span bridges. Chen et al. [7] proposed a framework for fatigue analysis of a long-span suspension bridge under railway, highway, and wind loading by integrating computer simulation with SHMSs, and it was applied to evaluate fatigue damage of the Tsing Ma Suspension Bridge over its design life as a case study. Based on this work, Chen et al. [8] proposed a framework for fatigue reliability analysis of long suspension bridges under multiple loading, in which the probabilistic models of railway, highway, and wind loading were established based on the measurement data acquired from the SHMS of the Tsing Ma Bridge. Wu et al. [9] proposed a reliability-based fatigue approach for slender long-span bridge, and the combined dynamic loading effects from wind and traffic as well as the associated uncertainties were considered. Based on the assumption that dynamic magnification related to vehicle dynamics can be neglected in long suspension bridges, Chen et al. [8] established a framework for fatigue reliability analysis. To account for different types of long-span bridges with the span length ranging from a few hundred to thousands of meters, Zhang et al. [10] proposed a more general framework which includes multiple random variables for the dynamic loads in a bridge's life cycle for the vehicle-bridge-wind dynamic system, such as road profile, vehicle speed, and wind velocity and direction, among other effects.

### 3.2. Assessment under Extreme Events

The aforementioned fatigue assessment mainly focuses on damage accumulation induced by stress fluctuations under normal operational condition in a long-term period. For long-span bridges, in addition to the normal operational conditions in which wind speeds are small or moderate and traffic scenarios are normal, there are some extreme event conditions. Extreme events may include complex traffic congestion on the bridge, coupled with moderate or even strong wind [11]. For example, severe traffic congestions may be formed on the bridge as a result of an evacuation or a partial blockage of driving lanes due to traffic accidents, construction, or maintenance. For hurricane evacuations, there are usually a lot of road vehicles passing through the bridge before the landfall of the hurricane while the wind speed may become pretty high already [131].

Although the excessive dynamic responses of the bridges under extreme events are rare, it is also critical since it may cause critical damage initiation or accumulation on some local bridge members. Furthermore, the extreme events (e.g., heavy traffic) may even trigger the collapse of the whole bridge by breaking the “weakest link,” especially when some hidden damage or design flaw has not been detected. One recent example is the Minnesota Bridge failure which occurred during rush hours with heavy traffic although traffic loads may not be the direct cause of failure. For slender long-span bridges, strong wind may also cause threats by working interactively with heavy traffic loads. Therefore, even though the extreme cases associated with congested traffic and/or windy weather may be relatively rare and the durations could be short, it is still important for bridge engineers to appropriately look into these unusual extreme events during structural design and life-time management of these critical infrastructures [11].

The dynamic performance of long-span bridges under combined actions of strong winds and running road vehicles has been studied by many researchers in recent years [17, 79, 81, 82, 132]. Most of them studied bridge dynamic performance under road traffic in which only one or several vehicles distributed in an assumed (usually uniform) pattern on long-span bridges were considered. Extreme events such as traffic congestion coupled with strong wind were out of concern in those studies. Recently, Wu and Chen [11] conducted a research on the assessment of long-span bridges under extreme events, which includes complex traffic congestion coupled with moderate or even strong wind. This study applied the cellular automaton (CA) traffic model to the simulation of the actual traffic flow through the bridge, defined representative scenarios for the extreme events, and numerically studied the bridge performance under these possible extreme events.

## 4. Conclusions and Recommendations

Dynamic responses of long-span bridges are often required for assessing the safety of these bridges and can be determined by numerical simulation technologies. This paper provides a detailed review of key issues involved in dynamic response analysis of long-span multiload bridges based on numerical simulation, including dynamic interactions between running trains and bridge, between running road vehicles and bridge, and between wind and bridge, and in the wind-vehicle-bridge coupled system. Then, the review work was conducted for engineering applications of newly developed numerical simulation technologies to safety assessment of long-span bridges, such as assessment of fatigue damage and assessment under extreme event condition. Although technologies for numerical simulation of dynamic responses of long-span multiload bridge have achieved great advances in past few decades and successfully applied to several important bridges, it is still far from reach its maturity and enable to take place of traditional field measurement. The existing problems and promising research efforts at least include the following aspects.

1. After multiple types of dynamic interactions being considered in the complex system, computational efficiency is a bottleneck problem for numerical simulation of dynamic response of a long-span bridge. Typically when multiple loads are involved, a large number of loading combinations for multiple loadings must be considered in the assessment.
2. It is rather complex for the time-depending wind loads acting on a long-span bridge and running vehicles, especially for the case of rapid change of wind loads, such as a train passing through a bridge tower or two trains passing each other. The aerodynamic characteristics of vehicles and the bridge under different loading scenarios can be determined through the wind tunnel testing and used in numerical simulation of dynamic responses of the bridge and vehicles.
3. It is a new trend to integrate numerical simulation technologies and measurement data of structural health monitoring systems (SHMSs) installed on long-span bridges, which makes the safety assessment of bridge structures more reliable. Measured structural responses could be used to validate numerical simulation approach, and measured loading information could be used for generating statistical or probabilistic models of multiple loads.
4. It is important to study dynamic responses of bridge structures under extreme events, such as congested traffic coupled with windy weather, which happens in a long-span bridge. For the assessment under extreme events using numerical simulation technologies, simulation of traffic flow and definition of representative scenarios of the extreme events are key issues.
5. It is necessary to consider the effects of typhoon winds on the safety assessment of long-span bridges in a reasonable way. Few researches do this mostly because a probabilistic distribution of wind speed and direction specifically for typhoons is required for assessment, but there are insufficient measured records to establish a reliable probabilistic typhoon wind model.

## References

[1] Doebling SW, Farrar CR, Prime MB (1998). A summary review of vibration-based damage identification methods. *Shock and Vibration Digest*.

[2] Sohn H, Farrar CR, Hunter NF, Worden K (2001). Structural health monitoring using statistical pattern recognition techniques. *Journal of Dynamic Systems, Measurement and Control*.

[3] Fan W, Qiao P (2011). Vibration-based damage identification methods: a review and comparative study. *Structural Health Monitoring*.

[4] Zhu XQ, Law SS (2007). Damage detection in simply supported concrete bridge structure under moving vehicular loads. *Journal of Vibration and Acoustics, Transactions of the ASME*.

[5] Li J, Law SS (2012). Damage identification of a target substructure with moving load excitation. *Mechanical Systems and Signal Processing*.

[6] Li J, Law SS, Hao H (2013). Improved damage identification in bridge structures subject to moving loads: numerical and experimental studies. *International Journal of Mechanical Sciences*.

[7] Chen ZW, Xu YL, Xia Y, Li Q, Wong KY (2011). Fatigue analysis of long-span suspension bridges under multiple loading: case study. *Engineering Structures*.

[8] Chen ZW, Xu YL, Wang XM (2012). SHMS-based fatigue reliability analysis of multiloading suspension bridges. *Journal of Structural Engineering-Asce*.

[9] Wu J, Chen SR, van de Lindt JW (2012). Fatigue assessment of slender long-span bridges: reliability approach. *Journal of Bridge Engineering*.

[10] Zhang W, Cai CS, Pan F (2013). Fatigue reliability assessment for long-span bridges under combined dynamic loads from winds and vehicles. *Journal of Bridge Engineering*.

[11] Wu J, Chen SR (2011). Probabilistic dynamic behavior of a long-span bridge under extreme events. *Engineering Structures*.

[12] Ko JM, Ni YQ (2005). Technology developments in structural health monitoring of large-scale bridges. *Engineering Structures*.

[13] Yi TH, Li HN, Sun HM (2013). Multi-stage structural damage diagnosis method based on, “energy-damage” theory. *Smart Structures and Systems*.

[14] Yi TH, Li HN, Gu M (2010). Full-scale measurements of dynamic response of suspension bridge subjected to environmental loads using GPS technology. *Science China Technological Sciences*.

[15] Yi TH, Li HN, Gu M (2013). Experimental assessment of high-rate GPS receivers for deformation monitoring of bridge. *Measurement: Journal of the International Measurement Confederation*.

[16] Ye XW, Ni YQ, Wong KY, Ko JM (2012). Statistical analysis of stress spectra for fatigue life assessment of steel bridges with structural health monitoring data. *Engineering Structures*.

[17] Chen ZW, Xu YL, Li Q, Wu DJ (2011). Dynamic stress analysis of long suspension bridges under wind, railway, and highway loadings. *Journal of Bridge Engineering*.

[18] Meisenholder SG, Weidlinger P (1974). Dynamic interaction aspects of cable-stayed guideways for high speed ground transportation. *American Society of Mechanical Engineers*.

[19] Mao QH (1989). *Research on the Highway Bridge Vibration Due to Moving Vehicles*.

[20] Xu YL, Ko JM, Yu Z (1997). Modal analysis of tower-cable system of Tsing Ma long suspension bridge. *Engineering Structures*.

[21] Guo W, Xia H, Xu Y-L (2007). Dynamic response of a long span suspension bridge and running safety of a train under wind action. *Frontiers of Architecture and Civil Engineering in China*.

[22] Liu TT, Xu YL, Zhang WS, Wong KY, Zhou HJ, Chan KWY (2009). Buffeting-induced stresses in a long suspension bridge: structural health monitoring oriented stress analysis. *Wind and Structures, An International Journal*.

[23] Wong KY (2003). Structural identification of Tsing Ma Bridge. *Transactions Hong Kong Institution of Engineers*.

[24] Xu YL, Li Q, Wu DJ, Chen ZW (2010). Stress and acceleration analysis of coupled vehicle and long-span bridge systems using the mode superposition method. *Engineering Structures*.

[25] Duan YF, Xu YL, Fei QG (2011). Advanced finite element model of Tsing Ma Bridge for structural health monitoring. *International Journal of Structural Stability and Dynamics*.

[26] Li ZX, Zhou TQ, Chan THT, Yu Y (2007). Multi-scale numerical analysis on dynamic response and local damage in long-span bridges. *Engineering Structures*.

[27] Zhang W, Cai CS, Pan F (2013). Finite element modeling of bridges with equivalent orthotropic material method for multi-scale dynamic loads. *Engineering Structures*.

[28] Timoshenko SP (1922). On the forced vibrations of bridges. *Philosophical Magazine*.

[29] Ayre RS, Ford G, Jacobsen LS (1950). Transverse vibration of a two-span beam under the action of a moving constant force. *Journal of Applied Mechanics*.

[30] Ayre RS, Jacobsen LS (1950). Transverse vibration of a two-span beam under the action of a moving alternating force. *Journal of Applied Mechanics*.

[31] Fryba L (1972). *Vibration of Solids and Structures under Moving Loads*.

[32] Wu J-S, Dai C-W (1987). Dynamic response of multispan nonuniform beam due to moving loads. *Journal of Structural Engineering*.

[33] Weaver W, Timoshenko SP, Young DH (1990). *Vibration Problems in Engineering*.

[34] Galdos NH, Schelling DR, Sahin MA (1993). Methodology for impact factor of horizontally curved box bridges. *Journal of Structural Engineering*.

[35] Gbadeyan JA, Oni ST (1995). Dynamic behaviour of beams and rectangular plates under moving loads. *Journal of Sound and Vibration*.

[36] Zheng DY, Cheung YK, Au FTK, Cheng YS (1998). Vibration of multi-span non-uniform beams under moving loads by using modified beam vibration functions. *Journal of Sound and Vibration*.

[37] Rao GV (2000). Linear dynamics of an elastic beam under moving loads. *Journal of Vibration and Acoustics, Transactions of the ASME*.

[38] Yang YB, Yau JD, Wu YS (2004). *Vehicle-Bridge Interaction Dynamic: with Applications to High-Speed Railways*.

[39] Ting EC, Genin J, Ginsberg JH (1974). A general algorithm for moving mass problems. *Journal of Sound and Vibration*.

[40] Sadiku S, Leipholz HHE (1987). On the dynamics of elastic systems with moving concentrated masses. *Ingenieur-Archiv*.

[41] Akin JE, Mofid M (1989). Numerical solution for response of beams with moving mass. *Journal of Structural Engineering*.

[42] Mahmoud MA, Abou Zaid MA (2002). Dynamic response of a beam with a crack subject to a moving mass. *Journal of Sound and Vibration*.

[43] Garg VK (1994). *Dynamics of Railway Vehicle Systems*.

[44] Wang T-L, Huang D (1992). Cable-stayed bridge vibration due to road surface roughness. *Journal of Structural Engineering*.

[45] Yang Y-B, Lin B-H (1995). Vehicle-bridge interaction analysis by dynamic condensation method. *Journal of Structural Engineering*.

[46] Yang YB, Yau JD, Hsu LC (1997). Vibration of simple beams due to trains moving at high speeds. *Engineering Structures*.

[47] Tabarrok B, Esmailzadeh E (2000). Induced vibration of bridges transversed by moving vehicles. *Transactions of the Canadian Society for Mechanical Engineering B*.

[48] Liu C, Wang T-L, Huang D (2001). Impact study for multi-girder bridge based on correlated road roughness. *Structural Engineering and Mechanics*.

[49] Chu KH, Garg VK, Wang TL (1986). Impact in railway prestressed concrete bridges. *Journal of Structural Engineering*.

[50] Wang T-L, Garg VK, Chu K-H (1991). Railway bridge/vehicle interaction studies with new vehicle model. *Journal of Structural Engineering*.

[51] Xia H, Xu YL, Chan THT (2000). Dynamic interaction of long suspension bridges with running trains. *Journal of Sound and Vibration*.

[52] Zhang Q-L, Vrouwenvelder A, Wardenier J (2001). Numerical simulation of train-bridge interactive dynamics. *Computers and Structures*.

[53] Xia H, Zhang N, de Roeck G (2003). Dynamic analysis of high speed railway bridge under articulated trains. *Computers and Structures*.

[54] Diana G, Cheli F, Collina A, Corradi R, Melzi S (2002). The development of a numerical model for railway vehicles comfort assessment through comparison with experimental measurements. *Vehicle System Dynamics*.

[55] Li Q, Xu YL, Wu DJ, Chen ZW (2010). Computer-aided nonlinear vehicle-bridge interaction analysis. *Journal of Vibration and Control*.

[56] Wiriyachai A, Chu KH, Garg VK (1982). Bridge impact due to wheel and track irregularities. *Journal of the Engineering Mechanics Division*.

[57] Fryba L (1996). *Dynamics of Railway Bridges*.

[58] Huang D, Wang T-L (1992). Impact analysis of cable-stayed bridges. *Computers and Structures*.

[59] Zhai WM (2007). *Vehicle-Track Coupling Dynamics*.

[60] Olsson M (1985). Finite element, modal co-ordinate analysis of structures subjected to moving loads. *Journal of Sound and Vibration*.

[61] Yang Y-B, Chang C-H, Yau J-D (1999). An element for analysing vehicle-bridge systems considering vehicle’s pitching effect. *International Journal for Numerical Methods in Engineering*.

[62] Yang YB, Wu YS (2001). A versatile element for analyzing vehicle-bridge interaction response. *Engineering Structures*.

[63] Au FTK, Wang JJ, Cheung YK (2001). Impact study of cable-stayed bridge under railway traffic using various models. *Journal of Sound and Vibration*.

[64] Sun YQ, Dhanasekar M (2002). A dynamic model for the vertical interaction of the rail track and wagon system. *International Journal of Solids and Structures*.

[65] Henchi K, Fafard M, Talbot M, Dhatt G (1998). An efficient algorithm for dynamic analysis of bridges under moving vehicles using a coupled modal and physical components approach. *Journal of Sound and Vibration*.

[66] Xu YL, Wang LY (2003). Analytical study of wind-rain-induced cable vibration: SDOF model. *Journal of Wind Engineering and Industrial Aerodynamics*.

[67] Biondi B, Muscolino G, Sofi A (2005). A substructure approach for the dynamic analysis of train-track-bridge system. *Computers and Structures*.

[68] Humar JL, Kashif AH (1995). Dynamic response analysis of slab-type bridges. *Journal of Structural Engineering*.

[69] Lou P, Zeng Q-Y (2004). Formulation of equations of vertical motion for vehicle-track-bridge system. *Journal of the China Railway Society*.

[70] Coussy O, Said M, van Hoove J-P (1989). The influence of random surface irregularities on the dynamic response of bridges under suspended moving loads. *Journal of Sound and Vibration*.

[71] Hwang ES, Nowak AS (1991). Simulation of dynamic load for bridges. *Journal of Structural Engineering*.

[72] Yang F, Fonder GA (1996). An iterative solution method for dynamic response of bridge-vehicles systems. *Earthquake Engineering and Structural Dynamics*.

[73] Zhai W, Cai Z (1997). Dynamic interaction between a lumped mass vehicle and a discretely supported continuous rail track. *Computers and Structures*.

[74] Zhai WM, Cai CB (2003). Train/track/bridge dynamic interactions: simulation and applications. *Vehicle System Dynamics*.

[75] Bruno D, Greco F, Lonetti P (2008). Dynamic impact analysis of long span cable-stayed bridges under moving loads. *Engineering Structures*.

[76] Song XD, Wu DJ, Li Q (2014). Dynamic impact analysis of double-tower cable-stayed maglev bridges using a simple model. *Journal of Bridge Engineering*.

[77] Wu Y-S, Yang Y-B (2003). Steady-state response and riding comfort of trains moving over a series of simply supported bridges. *Engineering Structures*.

[78] Antolin P, Zhang N, Goicolea JM, Xia H, Astiz MA, Oliva J (2013). Consideration of nonlinear wheel-rail contact forces for dynamic vehicle-bridge interaction in high-speed railways. *Journal of Sound and Vibration*.

[79] Guo WH, Xu YL (2001). Fully computerized approach to study cable-stayed bridge-vehicle interaction. *Journal of Sound and Vibration*.

[80] Xu YL, Guo WH (2003). Dynamic behaviour of high-sided road vehicles subject to a sudden crosswind gust. *Wind and Structures*.

[81] Xu YL, Guo WH (2003). Dynamic analysis of coupled road vehicle and cable-stayed bridge systems under turbulent wind. *Engineering Structures*.

[82] Cai CS, Chen SR (2004). Framework of vehicle-bridge-wind dynamic analysis. *Journal of Wind Engineering and Industrial Aerodynamics*.

[83] Chen SR, Wu J (2011). Modeling stochastic live load for long-span bridge based on microscopic traffic flow simulation. *Computers and Structures*.

[84] Paultre AV, Yang B, Bergman LA, Tan CA (1992). Bridge dynamics and dynamic amplification factors—a review of analytical and experimental findings. *Canadian Journal of Civil Engineering*.

[85] Honda H, Kajikawa Y, Kobori T (1982). Spectra of road surface roughness of bridges. *Journal of the Structural Division*.

[86] Inbanathan MJ, Wieland M (1987). Bridge vibrations due to vehicle moving over rough surface. *Journal of Structural Engineering*.

[87] Wang T-L, Huang D (1992). Cable-stayed bridge vibration due to road surface roughness. *Journal of Structural Engineering*.

[88] Chatterjee PK, Datta TK, Surana CS (1994). Vibration suspension bridges under vehicular movement. *Journal of Structural Engineering*.

[89] Chang D, Lee H (1994). Impact factors for simple-span highway girder bridges. *Journal of Structural Engineering*.

[90] Pan T-C, Li J (2002). Dynamic vehicle element method for transient response of coupled vehicle-structure systems. *Journal of Structural Engineering*.

[91] Dodds CJ, Robson JD (1973). The description of road surface roughness. *Journal of Sound Vibration*.

[92] Huang D, Wang T-L, Shahawy M (1993). Impact studies of multigirder concrete bridges. *Journal of Structural Engineering*.

[93] Davenport AG (1962). Buffeting of a suspension bridge by storm wind. *Journal of Structural Division*.

[94] Scanlan RH (1978). The action of flexible bridges under wind, I: flutter theory. *Journal of Sound and Vibration*.

[95] Ding Q, Lee PKK (2000). Computer simulation of buffeting actions of suspension bridges under turbulent wind. *Computers and Structures*.

[96] Boonyapinyo V, Miyata T, Yamada H (1999). Advanced aerodynamic analysis of suspension bridges by state-space approach. *Journal of Structural Engineering*.

[97] Chen Y-H, Li C-Y (2000). Dynamic response of elevated high-speed railway. *Journal of Bridge Engineering*.

[98] Chen X, Matsumoto M, Kareem A (2000). Time domain flutter and buffeting response analysis of bridges. *Journal of Engineering Mechanics*.

[99] Chen X, Kareem A (2001). Equivalent static wind loads for buffeting response of bridges. *Journal of Structural Engineering*.

[100] Chen SR, Cai CS (2003). Evolution of long-span bridge response to wind-numerical simulation and discussion. *Computers and Structures*.

[101] Xu YL, Xia H, Yan QS (2003). Dynamic response of suspension bridge to high wind and running train. *Journal of Bridge Engineering*.

[102] Guo A, Xu YL, Li H (2007). Dynamic performance of cable-stayed bridge tower with multi-stage pendulum mass damper under wind excitations-II: experiment. *Earthquake Engineering and Engineering Vibration*.

[103] Simiu E, Scanlan RH (1996). *Wind Effects on Structures*.

[104] Cao Y, Xiang H, Zhou Y (2000). Simulation of stochastic wind velocity field on long-span bridges. *Journal of Engineering Mechanics*.

[105] Shinozuka M, Jan C-M (1972). Digital simulation of random processes and its applications. *Journal of Sound and Vibration*.

[106] Shum KM (2004). *Lateral and torsional vibration control of long span bridge deck using novel tuned liquid column dampers [Ph.D. thesis]*.

[107] Lin YK, Yang JN (1983). Multimode bridge response to wind excitations. *Journal of Engineering Mechanics*.

[108] Lin YS (1978). *Self-Excited Bridge Motion in Turbulent Wind*.

[109] Lau DT, Cheung MS, Cheng SH (2000). 3D flutter analysis of bridges by spline finite-strip method. *Journal of Structural Engineering*.

[110] Balzer LA (1977). Atmospheric turbulence encountered by high-speed ground transport vehicles. *Journal of Mechanical Engineering Science *.

[111] Cooper RK (1984). Atmospheric turbulence with respect to moving ground vehicles. *Journal of Wind Engineering and Industrial Aerodynamics*.

[112] Baker CJ (1986). A simplified analysis of various types of wind-induced road vehicle accidents. *Journal of Wind Engineering and Industrial Aerodynamics*.

[113] Baker CJ (1987). Measures to control vehicle movement at exposed sites during windy periods. *Journal of Wind Engineering and Industrial Aerodynamics*.

[114] Baker CJ (1988). High sided articulated road vehicles in strong cross winds. *Journal of Wind Engineering and Industrial Aerodynamics*.

[115] Baker CJ (1991). Ground vehicles in high cross winds part I: steady aerodynamic forces. *Journal of Fluids and Structures*.

[116] Baker CJ (1991). Ground vehicles in high cross winds part II: unsteady aerodynamic forces. *Journal of Fluids and Structures*.

[117] Xu YL, Ding QS (2006). Interaction of railway vehicles with track in cross-winds. *Journal of Fluids and Structures*.

[118] Chen SR, Wu J (2010). Dynamic performance simulation of long-span bridge under combined loads of stochastic traffic and wind. *Journal of Bridge Engineering*.

[119] Li Y, Qiang S, Liao H, Xu YL (2005). Dynamics of wind-rail vehicle-bridge systems. *Journal of Wind Engineering and Industrial Aerodynamics*.

[120] Xu YL, Zhang N, Xia H (2004). Vibration of coupled train and cable-stayed bridge systems in cross winds. *Engineering Structures*.

[121] Guo WW, Xu YL, Xia H, Zhang WS, Shum KM (2007). Dynamic response of suspension bridge to typhoon and trains. II: numerical results. *Journal of Structural Engineering*.

[122] Li YL, Xiang HY, Wang B, Xu YL, Qiang SZ (2013). Dynamic analysis of wind-vehicle-bridge coupling system during the meeting of two trains. *Advances in Structural Engineering*.

[123] Dorigatti F, Sterling M, Rocchi D (2012). Wind tunnel measurements of crosswind loads on high sided vehicles over long span bridges. *Journal of Wind Engineering and Industrial Aerodynamics*.

[124] Zhu LD, Li L, Xu YL, Zhu Q (2012). Wind tunnel investigations of aerodynamic coefficients of road vehicles on bridge deck. *Journal of Fluids and Structures*.

[125] Han Y, Hu JX, Cai CS, Chen ZQ, Li CG (2013). Experimental and numerical studies of aerodynamic forces on vehicles and bridges. *Wind and Structures*.

[126] ASCE (1982). Committee on fatigue and fracture reliability of the committee on structural safety and reliability of the structural division, fatigue reliability 1–4. *Journal of Structural Engineering*.

[127] BS BS5400: part 10, code of practice for fatigue.

[128] BS BS7608: code of practice for fatigue design and assessment of steel structures.

[129] AASHTO (1990). *Guide Specifications for Fatigue Evaluation of Existing Steel Bridges*.

[130] AASHTO (2003). *Guide Manual for Condition Evaluation and Load and Resistance Factor Rating (LRFR) of Highway Bridges*.

[131] Chen SR, Cai CS, Wolshon B (2009). From normal operation to evacuation: Single-vehicle safety under adverse weather, topographic, and operational conditions. *Natural Hazards Review*.

[132] Chen SR, Cai CS (2007). Equivalent wheel load approach for slender cable-stayed bridge fatigue assessment under traffic and wind: feasibility study. *Journal of Bridge Engineering*.

